# Demographics and Risk Factors of Pediatric Pulmonary Hypertension Readmissions

**DOI:** 10.7759/cureus.18994

**Published:** 2021-10-23

**Authors:** Mukul Sehgal, Amod Amritphale, Shashank Vadayla, Madhuri Mulekar, Mansi Batra, Nupur Amritphale, Lynn A Batten, Rosa Vidal

**Affiliations:** 1 Pediatric Critical Care, University of South Alabama College of Medicine, Mobile, USA; 2 Cardiology, University of South Alabama College of Medicine, Mobile, USA; 3 Computational Analysis and Modelling, Louisiana Tech University, Ruston, USA; 4 Mathematics and Statistics, University of South Alabama, Mobile, USA; 5 Pediatrics, University of South Alabama College of Medicine, Mobile, USA; 6 Pediatric Cardiology, University of South Alabama College of Medicine, Mobile, USA

**Keywords:** pulmonary hypertension, pediatric intensive care unit (picu), invasive mechanical ventilation, risk factors, pediatric clinical cardiology, severe sepsis, hospital readmission rate, readmission rate 30 days, pediatric critical care usa

## Abstract

Background and Objectives: Pulmonary hypertension (PH) leads to significant morbidity and mortality in pediatric patients and increases the readmission rates for hospitalizations. This study evaluates the risk factors and comorbidities associated with an increase in 30-day readmissions among pediatric PH patients.

Methods: National Readmission Database (NRD) 2017 was searched for patients less than 18 years of age who were diagnosed with PH based on the International Classification of Diseases, 10th Revision (ICD-10). Statistical Package for the Social Sciences (SPSS) software v25.0 (IBM Corp., Armonk, NY) was used for statistical analysis.

Results: Of 5.52 million pediatric encounters, 10,501 patients met the selection criteria. The 30-day readmission rate of 14.43% (p < 0.001) was higher than hospitalizations from other causes {Odds Ratio (OR) 4.02 (3.84-4.20), p < 0.001}. The comorbidities of sepsis {OR 0.75 (0.64-0.89), p < 0.02} and respiratory infections {OR 0.75 (0.67-0.85), p < 0.001} were observed to be associated with lower 30-day readmissions. Patients who required invasive mechanical ventilation via endotracheal tube {OR 1.66 (1.4-1.96), p < 0.001} or tracheostomy tube {OR 1.35 (1.15-1.6), p < 0.001} had increased unplanned readmissions. Patients with higher severity of illness based on All Patients Refined Diagnosis Related Groups (APR-DRG) were more likely to get readmitted {OR 7.66 (3.13-18.76), p < 0.001}.

Conclusion: PH was associated with increased readmission rates compared to the other pediatric diagnoses, but the readmission rate in this study was lower than one previous pediatric study. Invasive mechanical ventilation, Medicaid insurance, higher severity of illness, and female gender were associated with a higher likelihood of readmission within 30 days.

## Introduction

Pulmonary hypertension (PH) has been defined as pulmonary arterial pressures greater than 25 mmHg at rest, as per the first World Symposium on Pulmonary Hypertension (WSPH) organized by the World Health Organization (WHO) in Geneva in 1973 [1]. Pediatric PH differs in terms of pathophysiology and etiology as compared to adults [2]. Knowledge acquired over the last decade about pediatric PH is now included in the clinical classification of PH [3]. PH in pediatric patients is associated with significant morbidity and mortality [4]. These patients have various comorbidities, with the most common being congenital heart disease (CHD), bronchopulmonary dysplasia (BPD), genetic anomalies affecting the lung, and pathological insults on growing lungs [5,6]. While the number of hospitalizations for PH has increased over time, there has been a decrease in mortality of these patients, accompanied by a considerable increase in hospital charges [4].

Hospital readmission is one of the quality indicators tracked by the Centers for Medicare & Medicaid Services (CMS) and a target for hospital cost saving as per the Patient Protection and Affordable Care Act [7]. Many states have Medicaid readmission penalties in the pediatric population that focus on overall readmission rates [8,9]. PH is associated with increased hospital readmissions in both children and adults [10,11]. According to Awerbach et al. (2018), the 30-day readmission rate in pediatric patients with PH was 26.3% and was associated with increased odds in those with public insurance [11]. This study highlights readmission rates and other covariates using Pediatric Health Information System (PHIS) among patients in tertiary care hospitals.

Current evidence suggests that readmission rates can be improved if the patients who are at higher risk for readmission are identified in a timely manner, and necessary measures are taken to reduce the deleted readmission. These measures include proper discharge planning, patient education, and timely outpatient appointments [12].

## Materials and methods

Study design

A retrospective study was performed using an all-patient publicly available database for the year 2017. Diagnosis codes pertaining to PH were used to identify index admissions. Our study was exempted from the Institutional Review Board (IRB) at the University of South Alabama as we used a publicly available database.

Data source

We obtained data using the National Readmission Database (NRD) from January 1, 2017, to December 1, 2017. NRD is the largest all-patient, all-payer inpatient database available in the United States. The NRD is developed and maintained by the Agency for Healthcare Research and Quality for the Healthcare Cost and Utilization Project (HCUP) and includes publicly available hospitalization data from nonfederal hospitals. The 2017 NRD includes data from 28 states and approximately five million pediatric discharges. It represents 58.2% of all hospitalizations in the year 2017 and 60% of the population. Unique patient identifiers are allotted to each admission to allow for tracking readmissions within a state in a given year. Moreover, data regarding patient and hospital demographics as well as diagnostic and procedural codes assigned at the time of discharge are also available.

Inclusion criteria included patients less than 18 years of age, who had a primary diagnosis of PH using the International Classification of Diseases, 10th Revision, Clinical Modification (ICD-10-CM) codes (Appendix A).

Exclusion criteria included the following: (1) age greater than 18 years, (2) initial visits in December 2017 because they could not be followed beyond 30 days, (3) admitted electively on the readmission visit, (4) no patient encounters within 30 days from the primary visit, (5) transferred out for rehabilitation services, and (6) died on the index admission.

Covariates

Patient demographics for the index admission including age, gender, insurance status, median household income, loss of function, and discharge disposition were collected. Comorbidities were also included using ICD-10-CM codes (Appendices B-F). Hospital demographics such as bed size, ownership (government versus private), designation (large, small, micropolitan, non-urban), and teaching status were also obtained. Various procedures done during the hospitalization were identified using ICD-10 procedure codes (Appendices G-I). The severity of illness was estimated using All Patients Refined Diagnosis Related Group (APR-DRG) (Appendix J).

Statistical analysis

All statistical analysis was performed using IBM SPSS Statistics for Windows, version 1.0.0.1327 (IBM Corp., Armonk, USA). Baseline characteristics of participants such as age, gender, weekend versus weekday admissions, household income, payer status, loss of function, and the likelihood of dying were tested for statistical differences using the Pearson chi-square test for categorical variables and Mann-Whitney U-Test for continuous variables with no readmission as the reference group. Clinical predictors for 30-day readmission were analyzed using multivariable logistic regression. Furthermore, multiple logistic regressions were performed to independently determine the predictors of readmission within each clinical variable with values presented as Odds Ratios (OR) with 95% confidence intervals (CI).

## Results

Of 5,529,389 pediatric hospital encounters in NRD, 16,590 encounters were for PH. Among patients with PH, 748 (7.1%) patients died during the index admission and were excluded from the study. Infants comprised the majority of deaths with 577 (77.1%). Comparison of severity of illness using APR-DRG severity score showed that among infants who died, 475 (82.4%) were classified as extreme loss of function compared to 66.3% among infants who survived (p < 0.001).

A total of 10,501 unique patients met the selection criteria of which 14.4% were readmitted within 30 days. The 30-day readmission rate for PH was higher than that from all other causes combined {Odds Ratio (OR) 4.02 (3.84-4.20), p < 0.001}. Among all PH patients, 56.6% were infants, who were found to have decreased odds of 30-day readmission as compared to the other age groups {OR 0.72 (0.65-0.80) p < 0.001}. For patients readmitted in less than 30 days, the average time to readmission was 17 days (±7 days).

Overall, lengths of stay for readmissions were significantly shorter than those for the initial admissions (median four days vs. nine days, p < 0.001). As a result, readmissions were significantly less expensive than the initial visits (median $36,313 vs. $85,121, p < 0.001). In 2017, total charges for all pediatric PH patient encounters in the United States amounted to $1.63 billion, while charges for 30-day unplanned readmissions for PH were $328 million in 2017. Medicaid remained the largest payer (63.9%) among all PH admissions (Table 1).

**Table 1:** Demographic characteristics of the sample Comparison of various demographic characteristics was done using chi-square analysis. IQR, interquartile range.

	No Readmission	30-Day Readmissions	p-Value
Number of Patients	8985 (85.56%)	1516 (14.43%)	<0.001
Age {IQR}	<1 year {0-3}	1 year {0-3}	
Age < 1 (%)	5189 (57.8)	755 (49.8)	
Age 1-7 years (%)	2391 (26.6)	501 (33)	
Age 8-13 years (%)	600 (6.7)	140 (9.2)	
Age 14-18 (%)	804 (8.9)	120 (7.9)	
Length of stay {Median (IQR)}	9 days {3-27}	9 days {4-24}	0.441
Total charges {Median (IQR)}	$98,433 (29,509-306,504)	$85,121 (34,485-296,303)	0.015
Female (%)	4082 (45.4)	776 (51.2)	<0.001
Payer (%)			
Medicaid	5474 (60.9)	1056 (69.6)	
Medicare	26 (0.3)	0 (0)	
Private insurance	3131 (34.8)	395 (26)	
Self-pay	115 (1.3)	5 (0.3)	
No charge	11 (0.1)	56 (3.7)	
Other	225 (2.5)	3 (0.2)	
Teaching status of the hospital (%)			
Metropolitan teaching	8061 (89.7)	1383 (91.2)	0.133
Metropolitan non-teaching	806 (9)	119 (7.8)	
Non-metropolitan hospital	118 (1.3)	14 (0.9)	
Location of the hospital (%)			
Large metropolitan areas with at least one million residents	5840 (65)	1131 (74.6)	<0.001
Small metropolitan areas with less than one million residents	3028 (33.7)	371 (24.5)	
Micropolitan areas	107 (1.2)	14 (0.9)	
Not metropolitan or micropolitan (non-urban residual)	11 (0.1)	0 (0)	
Median household incomes (%)			
$1-$43,999	2733 (30.4)	501 (33.1)	0.920
$44,000-$55,999	2397 (26.7)	413 (27.3)	
$56,000-$73,999	2405 (26.8)	386 (25.5)	
>$74,000	1375 (15.3)	212 (14)	

CHD was present in 52.9% of the patients with PH, while BPD was found in 17.6% of the patients of which 71.6% were infants. Other common comorbidities included respiratory infections (29.2%) and sepsis (6%). Among PH patients, 7.6% required extracorporeal membrane oxygenation (ECMO) during the initial visit, 17.7% required intubation, while 14.9% required mechanical ventilation using a tracheostomy tube (Table 2).

**Table 2:** Multivariate logistic regression analysis to predict 30-day readmission The comparison of various risk factors and comorbidities was done using logistic regression. APR-DRG, All Patients Refined Diagnosis Related Group; ECMO, extracorporeal membrane oxygenation.

Risk Factors	Odds Ratio (95% CI)	p-Value
Sepsis	0.75 (0.64-0.89)	<0.001
Mechanical ventilation with intubation	1.66 (1.4-1.96)	<0.001
Mechanical ventilation with tracheostomy tube	1.35 (1.15-1.6)	<0.001
ECMO	1.21 (0.97-1.52)	0.09
Respiratory infection	0.75 (0.67-0.85)	<0.001
Bronchopulmonary dysplasia	1.11 (0.96-1.28)	0.17
Congenital heart disease	0.99 (0.88-1.11)	0.87
Nitric oxide	0.78 (0.55-1.11)	0.17
Age		
Age < 1	Reference	
Age 1-7 years	1.29 (1.12-1.48)	<0.001
Age 8-13 years	1.44 (1.16-1.79)	<0.001
Age 14-18 years	1.01 (0.8-1.26)	0.96
Payer		
Medicaid	Reference	
Medicare	0 (0-)	1.00
Private insurance	0.7 (0.61-0.79)	<0.001
Self-pay	0.26 (0.11-0.63)	<0.001
No charge	0 (0-)	1.00
Other	1.27 (0.94-1.72)	0.13
Teaching status of the hospital		
Metropolitan teaching	Reference	
Metropolitan non-teaching	0.88 (0.72-1.08)	0.23
Location of the hospital		
Large metropolitan areas with at least one million residents	Reference	
Small metropolitan areas with less than one million residents	0.64 (0.57-0.73)	<0.001
Micropolitan areas	0.72 (0.41-1.28)	0.27
APR-DRG severity		
Minor loss of function (includes cases with no comorbidity or complications)	Reference	
Moderate loss of function	3.76 (1.49-9.47)	<0.001
Major loss of function	5.82 (2.37-14.25)	<0.001
Extreme loss of function	7.66 (3.13-18.76)	<0.001

Hospitals located in metropolitan areas with a population of less than one million were less likely to have 30-day readmissions for PH compared to those located in more populous areas {OR 0.62 (0.55-0.71), p < 0.001}. A comparison of teaching hospitals in large and small metropolitan areas showed that the former had higher rates of 30-day readmissions {OR 1.48 (1.37-1.68), p < 0.001}. However, it should be noted that compared to teaching hospitals from the small metro areas, those in the large metro areas were more likely to have patients with major or extreme loss of function as defined by categories 3 and 4, respectively, of APR-DRG {OR 1.28 (1.12-1.3), p < 0.001}, which may have resulted in the higher rate of 30-day readmissions.

## Discussion

The pediatric PH 30-day readmission rate in this study was 14.4% (Table 1). This is very similar to the readmission rate of 14.7%, which was reported in a study of adults by Chatterjee et al. based on the NRD 2013 database. However, Awerbach’s 2018 study based on the PHIS database showed that readmission in pediatric PH was higher (at 26.3%) [11]. This may be due to the acuity of patients in the PHIS study [11,13]. The PHIS database includes more than 40 tertiary care children’s hospitals with most of them located in metropolitan areas with a population > one million, which in our study was associated with more patients having extreme loss of function APR-DRG severity compared to other levels of severity [14]. Since one-third of patients in our study were admitted to hospitals located in smaller metropolitan areas (population < one million), we sampled a more comprehensive population distribution as compared to the PHIS database, which may explain the lower readmission rate observed in this study (Table 1).

Similar to the PHIS study, a majority of PH cases in this study were associated with CHD, followed by BPD; similar results have been reported by other studies [15]. However, there has been an improvement in morbidity and mortality of these patients in the last few decades due to the advent of newer treatment modalities and early surgical repair of CHD [16]. This could also be a reason that while PH was present in many patients with CHD, it was not associated with increased readmission rates. In our study, both sepsis and respiratory infections were found to be associated with decreased readmissions in PH patients (Table 2). Although several pediatric studies have shown sepsis to be a risk factor in increased readmissions as well as an independent risk factor in increased morbidity and mortality in pediatric populations, our study did not show similar results [17-19]. A possible explanation could be due to transient PH in patients with sepsis; therefore, once the infection resolves during the initial admission, these patients were less likely to be readmitted than those without sepsis as a trigger [20]. Similarly, transient PH can occur in the setting of respiratory infections [21].

**Figure 1:**
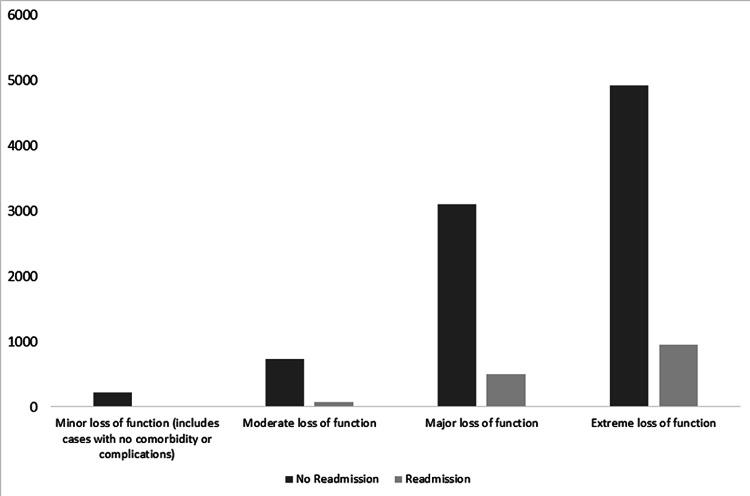
Outcome of the index visit based on the severity of illness The severity of illness is based on All Patients Refined Diagnosis Related Group (APR-DRG) severity of illness scoring, and the difference between the two groups was found to be statistically significant (p < 0.001).

This study demonstrates that patients requiring invasive ventilation via either an endotracheal tube or tracheostomy tube had higher odds of readmission (Table 2) as compared to those who did not. Studies have shown mechanical ventilation as an independent risk factor for readmission [22,23]. The need for mechanical ventilation in patients with PH is associated with increased morbidity and mortality [24]. Similarly, the severity of illness based on APR-DRG shows that patients with extreme loss of function during the initial visit are more likely to be readmitted (Figure 1). Studies have shown that APR-DRG is an effective way to measure morbidity and mortality in ICUs and to correlate well with physiological scores for the same [25,26].

Similar to other studies, this study showed that patients with Medicaid as their primary insurance had a higher likelihood of readmission [27,28]. A study in the adult population has shown that about half of the Medicaid patients did not have an outpatient follow-up between their initial visit and unplanned 30-day readmission [29]. We also found that the average time for PH readmissions was 17 days (±7 days); therefore, outpatient visits should be scheduled in 10-24 days after discharge from inpatient admissions to prevent readmissions effectively.

Study limitations

This study has a few limitations. First, in an administrative database from which study data is derived, the accuracy is only as good as the codes entered. Therefore, like any other administrative database, there is a possibility that some disease processes may have been coded inaccurately. Second, there may be factors not recorded in a database that might contribute to readmissions; hence, a study of those factors could also help in predicting these readmissions in the future. Third, individual patient severity of illness scores is not possible in a database; thus, the calculation of severity of illness using the APR-DRG may have inherent inaccuracies.

## Conclusions

This study demonstrates that while PH is associated with significant overall readmissions in pediatrics, the readmission rate is lower than that reported by the previous research. We found that patients with sepsis and respiratory infections during the initial visit were less likely to get readmitted within 30 days. Patients who required mechanical ventilation either via an endotracheal tube or tracheostomy tube were more likely to get readmitted. We found that the average time to readmission was 17 days, a finding that suggests that follow-up visits should be scheduled within two weeks of discharge, thereby potentially preventing some readmissions.

## Appendices

Appendix A 

**Table 3:** Pulmonary hypertension – ICD-10 codes ICD-10, International Classification of Diseases, 10th Revision.

I270	Primary pulmonary hypertension
I272	Other secondary pulmonary hypertension
I2720	Pulmonary hypertension, unspecified
I2721	Secondary pulmonary arterial hypertension
I2722	Pulmonary hypertension due to left heart disease
I2723	Pulmonary hypertension due to lung diseases and hypoxia
I2724	Chronic thromboembolic pulmonary hypertension
I2729	Other secondary pulmonary hypertension
I2781	Cor pulmonale (chronic)
I2782	Chronic pulmonary embolism
I2783	Eisenmenger syndrome
I289	Disease of pulmonary vessels, unspecified

Appendix B 

**Table 4:** Congenital heart disease – Clinical Classification System (CCS)

Q200	Common arterial trunk
Q201	Double outlet right ventricle
Q202	Double outlet left ventricle
Q203	Discordant ventriculoarterial connection
Q204	Double inlet ventricle
Q205	Discordant atrioventricular connection
Q206	Isomerism of atrial appendages
Q208	Other congenital malformation of cardiac chambers and connections
Q209	Congenital malformation of cardiac chambers and connections, unspecified
Q210	Ventricular septal defect
Q211	Atrial septal defect
Q212	Atrioventricular septal defect
Q213	Tetralogy of Fallot
Q214	Aortopulmonary septal defect
Q218	Other congenital malformations of cardiac septa
Q219	Congenital malformation of cardiac septum, unspecified
Q220	Pulmonary valve atresia
Q221	Congenital pulmonary valve stenosis
Q222	Congenital pulmonary valve insufficiency
Q223	Other congenital malformations of pulmonary valve
Q224	Congenital tricuspid stenosis
Q225	Ebstein anomaly
Q226	Hypoplastic right heart syndrome
Q228	Other congenital malformations of tricuspid valve
Q229	Congenital malformation of tricuspid valve, unspecified
Q230	Congenital stenosis of aortic valve
Q231	Congenital insufficiency of aortic valve
Q232	Congenital mitral stenosis
Q233	Congenital mitral insufficiency
Q234	Hypoplastic left heart syndrome
Q238	Other congenital malformations of aortic and mitral valves
Q239	Congenital malformation of aortic and mitral valves, unspecified
Q240	Dextrocardia
Q241	Levocardia
Q242	Cor triatriatum
Q243	Pulmonary infundibular stenosis
Q244	Congenital subaortic stenosis
Q245	Malformation of coronary vessels
Q246	Congenital heart block
Q248	Other specified congenital malformations of heart
Q249	Congenital malformation of heart, unspecified
Q250	Patent ductus arteriosus
Q251	Coarctation of aorta
Q252	Atresia of aorta
Q2521	Interruption of aortic arch
Q2529	Other atresia of aorta
Q253	Supravalvular aortic stenosis
Q254	Other congenital malformations of aorta
Q2540	Congenital malformation of aorta, unspecified
Q2541	Absence and aplasia of aorta
Q2542	Hypoplasia of aorta
Q2543	Congenital aneurysm of aorta
Q2544	Congenital dilation of aorta
Q2545	Double aortic arch
Q2546	Tortuous aortic arch
Q2547	Right aortic arch
Q2548	Anomalous origin of subclavian artery
Q2549	Other congenital malformations of aorta
Q255	Atresia of pulmonary artery
Q256	Stenosis of pulmonary artery
Q2571	Coarctation of pulmonary artery
Q2572	Congenital pulmonary arteriovenous malformation
Q2579	Other congenital malformations of pulmonary artery
Q258	Other congenital malformations of other great arteries
Q259	Congenital malformation of great arteries, unspecified
Q260	Congenital stenosis of vena cava
Q261	Persistent left superior vena cava
Q262	Total anomalous pulmonary venous connection
Q263	Partial anomalous pulmonary venous connection
Q264	Anomalous pulmonary venous connection, unspecified
Q265	Anomalous portal venous connection
Q266	Portal vein-hepatic artery fistula
Q268	Other congenital malformations of great veins
Q269	Congenital malformation of great vein, unspecified
Q270	Congenital absence and hypoplasia of umbilical artery
Q271	Congenital renal artery stenosis
Q272	Other congenital malformations of renal artery
Q2730	Arteriovenous malformation, site unspecified
Q2731	Arteriovenous malformation of vessel of upper limb
Q2732	Arteriovenous malformation of vessel of lower limb
Q2733	Arteriovenous malformation of digestive system vessel
Q2734	Arteriovenous malformation of renal vessel
Q2739	Arteriovenous malformation, other site
Q274	Congenital phlebectasia
Q278	Other congenital malformations of the peripheral vascular system
Q279	Congenital malformation of peripheral vascular system, unspecified
Q280	Arteriovenous malformation of precerebral vessels
Q281	Other malformations of precerebral vessels
Q282	Arteriovenous malformation of cerebral vessels
Q283	Other malformations of cerebral vessels
Q288	Other congenital malformations of the circulatory system
Q289	Congenital malformation of circulatory system, unspecified

Appendix C

International Classifications of Disease (ICD-10) and Clinical Classification Software (CCS) are used for this paper.

**Table 5:** Sepsis – Clinical Classification of Disease

'A021'	Salmonella sepsis
'A207'	Septicemic plague
'A227'	Anthrax sepsis
'A267'	Erysipelothrix sepsis
'A327'	Listerial sepsis
'A392'	Acute meningococcemia
'A393'	Chronic meningococcemia
'A394'	Meningococcemia, unspecified
'A400'	Sepsis due to streptococcus, group A
'A401'	Sepsis due to streptococcus, group B
'A403'	Sepsis due to *Streptococcus pneumoniae*
'A408'	Other streptococcal sepsis
'A409'	Streptococcal sepsis, unspecified
'A4101'	Sepsis due to methicillin-susceptible *Staphylococcus aureus*
'A4102'	Sepsis due to methicillin-resistant* Staphylococcus aureus*
'A411'	Sepsis due to other specified staphylococcus
'A412'	Sepsis due to unspecified staphylococcus
'A413'	Sepsis due to *Hemophilus influenzae*
'A414'	Sepsis due to anaerobes
'A4150'	Gram-negative sepsis, unspecified
'A4151'	Sepsis due to* Escherichia coli* (*E. coli*)
'A4152'	Sepsis due to Pseudomonas
'A4153'	Sepsis due to Serratia
'A4159'	Other Gram-negative sepsis
'A4181'	Sepsis due to Enterococcus
'A4189'	Other specified sepsis
'A419'	Sepsis, unspecified organism
'A427'	Actinomycotic sepsis
'A5486'	Gonococcal sepsis
'B007'	Disseminated herpesviral disease
'B377'	Candidal sepsis
'I76'	Septic arterial embolism
'R6520'	Severe sepsis without septic shock
'R6521'	Severe sepsis with septic shock

Appendix D 

**Table 6:** Respiratory infections – Clinical Classification of Disease

'A0103'	Typhoid pneumonia
'A0222'	Salmonella pneumonia
'A202'	Pneumonic plague
'A212'	Pulmonary tularemia
'A221'	Pulmonary anthrax
'A310'	Pulmonary mycobacterial infection
'A3791'	Whooping cough, unspecified species with pneumonia
'A430'	Pulmonary nocardiosis
'A481'	Legionnaires' disease
'B012'	Varicella pneumonia
'B052'	Measles complicated by pneumonia
'B0681'	Rubella pneumonia
'B250'	Cytomegaloviral pneumonitis
'B371'	Pulmonary candidiasis
'B380'	Acute pulmonary coccidioidomycosis
'B381'	Chronic pulmonary coccidioidomycosis
'B382'	Pulmonary coccidioidomycosis, unspecified
'B390'	Acute pulmonary histoplasmosis capsulati
'B391'	Chronic pulmonary histoplasmosis capsulati
'B392'	Pulmonary histoplasmosis capsulati, unspecified
'B583'	Pulmonary toxoplasmosis
'B59'	Pneumocystosis
'B7781'	Ascariasis pneumonia
'J120'	Adenoviral pneumonia
'J121'	Respiratory syncytial virus pneumonia
'J122'	Parainfluenza virus pneumonia
'J123'	Human metapneumovirus pneumonia
'J1281'	Pneumonia due to SARS-associated coronavirus
'J1289'	Other viral pneumonia
'J129'	Viral pneumonia, unspecified
'J13'	Pneumonia due to* Streptococcus pneumoniae*
'J14'	Pneumonia due to *Hemophilus influenzae*
'J150'	Pneumonia due to *Klebsiella pneumoniae*
'J151'	Pneumonia due to Pseudomonas
'J1520'	Pneumonia due to Staphylococcus, unspecified
'J15211'	Pneumonia due to methicillin-susceptible *Staphylococcus aureus*
'J15212'	Pneumonia due to methicillin-resistant *Staphylococcus aureus*
'J1529'	Pneumonia due to other Staphylococcus
'J153'	Pneumonia due to Streptococcus, group B
'J154'	Pneumonia due to other Streptococci
'J155'	Pneumonia due to Escherichia coli
'J156'	Pneumonia due to other Gram-negative bacteria
'J157'	Pneumonia due to *Mycoplasma pneumoniae*
'J158'	Pneumonia due to other specified bacteria
'J159'	Unspecified bacterial pneumonia
'J160'	Chlamydial pneumonia
'J168'	Pneumonia due to other specified infectious organisms
'J17'	Pneumonia in diseases classified elsewhere
'J180'	Bronchopneumonia, unspecified organism
'J181'	Lobar pneumonia, unspecified organism
'J188'	Other pneumonia, unspecified organism
'J189'	Pneumonia, unspecified organism
'J851'	Abscess of lung with pneumonia
'J200'	Acute bronchitis due to *Mycoplasma pneumoniae*
'J201'	Acute bronchitis due to *Hemophilus influenzae*
'J202'	Acute bronchitis due to Streptococcus
'J203'	Acute bronchitis due to Coxsackievirus
'J204'	Acute bronchitis due to Parainfluenza virus
'J205'	Acute bronchitis due to respiratory syncytial virus
'J206'	Acute bronchitis due to rhinovirus
'J207'	Acute bronchitis due to echovirus
'J208'	Acute bronchitis due to other specified organisms
'J209'	Acute bronchitis, unspecified
'J210'	Acute bronchiolitis due to respiratory syncytial virus
'J211'	Acute bronchiolitis due to human metapneumovirus
'J218'	Acute bronchiolitis due to other specified organisms
'J219'	Acute bronchiolitis, unspecified
'J40'	Bronchitis, not specified as acute or chronic
'A360'	Pharyngeal diphtheria
'A361'	Nasopharyngeal diphtheria
'A362'	Laryngeal diphtheria
'B085'	Enteroviral vesicular pharyngitis
'J00'	Acute nasopharyngitis (common cold)
'J020'	Streptococcal pharyngitis
'J028'	Acute pharyngitis due to other specified organisms
'J029'	Acute pharyngitis, unspecified
'J040'	Acute laryngitis
'J0410'	Acute tracheitis without obstruction
'J0411'	Acute tracheitis with obstruction
'J042'	Acute laryngotracheitis
'J0430'	Supraglottitis, unspecified, without obstruction
'J0431'	Supraglottitis, unspecified, with obstruction
'J050'	Acute obstructive laryngitis (croup)
'J0510'	Acute epiglottitis without obstruction
'J0511'	Acute epiglottitis with obstruction
'J060'	Acute laryngopharyngitis
'J069'	Acute upper respiratory infection, unspecified
'J390'	Retropharyngeal and parapharyngeal abscess
'J391'	Other abscess of pharynx

Appendix E 

**Table 7:** Acute renal failure – CCS CCS, Clinical Classification System.

'N170'	Acute Kidney Failure With Tubular Necrosis
'N171'	Acute kidney failure with acute cortical necrosis
'N172'	Acute kidney failure with medullary necrosis
'N178'	Other acute kidney failure
'N179'	Acute kidney failure, unspecified
'N19'	Unspecified kidney failure

Appendix F 

**Table 8:** Chronic renal failure - CCS CCS, Clinical Classification System.

'I120'	Hypertensive Chronic Kidney Disease With Stage 5 Chronic Kidney Disease or End-Stage Renal Disease
'I129'	Hypertensive chronic kidney disease, with stage 1 through stage 4 chronic kidney disease, or unspecified chronic kidney disease
'I1310'	Hypertensive heart and chronic kidney disease without heart failure, with stage 1 through stage 4 chronic kidney disease, or unspecified chronic kidney disease
'I1311'	Hypertensive heart and chronic kidney disease without heart failure, with stage 5 chronic kidney disease, or end-stage renal disease
'I132'	Hypertensive heart and chronic kidney disease with heart failure and with stage 5 chronic kidney disease, or end-stage renal disease
'N181'	Chronic kidney disease, stage 1
'N182'	Chronic kidney disease, stage 2 (mild)
'N183'	Chronic kidney disease, stage 3 (moderate)
'N184'	Chronic kidney disease, stage 4 (severe)
'N185'	Chronic kidney disease, stage 5
'N186'	End-stage renal disease
'N189'	Chronic kidney disease, unspecified

Appendix G 

**Table 9:** Mechanical ventilation – ICD-10 codes ICD-10, International Classification of Diseases, 10th Revision; CPAP, continuous positive airway pressure.

'09HN7BZ'	Insertion of Airway into Nasopharynx, via Opening
'09HN8BZ'	Insertion of airway into nasopharynx, endo
'0BH13EZ'	Insertion of endotracheal airway into trachea, percutaneous approach
'0BH17EZ'	Insertion of endotracheal airway into trachea, via opening
'0BH18EZ'	Insertion of endotracheal airway into trachea, endo
'0CHY7BZ'	Insertion of airway into mouth and throat, via opening
'0CHY8BZ'	Insertion of airway into mouth and throat, endo
'0DH57BZ'	Insertion of airway into esophagus, via opening
'0DH58BZ'	Insertion of airway into esophagus, endo
'0WHQ73Z'	Insertion of infusion device into respiratory tract, via opening
'0WHQ7YZ'	Insertion of other device into respiratory tract, via opening
'5A09357'	Assistance with respiratory ventilation, <24 hours, CPAP
'5A09457'	Assistance with respiratory ventilation, 24-96 hours, CPAP
'5A09557'	Assistance with respiratory ventilation, >96 hours, CPAP
'5A1935Z'	Respiratory ventilation, less than 24 consecutive hours
'5A1945Z'	Respiratory ventilation, 24-96 consecutive hours

Appendix H 

**Table 10:** ECMO ICD-10 codes ECMO, Extracorporeal membrane oxygenation; ICD-10, International Classification of Diseases, 10th Revision.

'5A15223'	Extracorporeal Membrane Oxygenation Continuous
'5A1522F'	Extracorporeal oxygenation, membrane, central
'5A1522G'	Extracorporeal oxygenation, peripheral VA ECMO
'5A1522H'	Extracorporeal oxygenation, membrane, peripheral veno-venous
'5A15A2F'	Extracorporeal oxygenation, membrane, central, intraoperatively
'5A15A2G'	Extracorporeal oxygenation, peripheral VA ECMO, intraoperatively
'5A15A2H'	Extracorporeal oxygenation, peripheral VV ECMO, intraoperatively

Appendix I 

**Table 11:** Other ICD-10 codes used that are not a part of CCS ICD-10, International Classification of Diseases, 10th Revision; CCS, Clinical Classification System.

Bronchopulmonary dysplasia	P27.1
Intubation	09HN7BZ, 09HN8BZ, 0BH13EZ, 0BH17EZ, 0BH18EZ, 0CHY7BZ, 0CHY8BZ

Appendix J 

**Table 12:** All Patients Refined Diagnosis Related Group (APR-DRG): severity of illness

Severity Level	Description
0	No class specified
1	Minor loss of function (includes cases with no comorbidity or complications)
2	Moderate loss of function
3	Major loss of function
4	Extreme loss of function
